# Naltrexone plus bupropion combination medication maintenance treatment for binge-eating disorder following successful acute treatments: randomized double-blind placebo-controlled trial

**DOI:** 10.1017/S0033291723001800

**Published:** 2023-12

**Authors:** Carlos M. Grilo, Janet A. Lydecker, Ralitza Gueorguieva

**Affiliations:** 1 Department of Psychiatry, Yale University School of Medicine, New Haven, CT, USA; 2 Department of Biostatistics, Yale School of Public Health, New Haven, CT, USA

**Keywords:** Binge eating, eating disorders, maintenance, obesity, pharmacotherapy, weight loss

## Abstract

**Background:**

Certain treatments have demonstrated acute efficacy for binge-eating disorder (BED) but there is a dearth of controlled research examining pharmacotherapies as maintenance treatments for responders to initial interventions. This gap in the literature is particularly critical for pharmacotherapy for BED which is associated with relapse following discontinuation. The current study tested the efficacy of naltrexone/bupropion maintenance treatment amongst responders to acute treatments for BED.

**Methods:**

Prospective randomized double-blind placebo-controlled single-site trial, conducted August 2017–December 2021, tested naltrexone/bupropion as maintenance treatment for responders to acute treatments with naltrexone/bupropion and/or behavioral weight-loss therapy for BED with comorbid obesity. Sixty-six patients (84.8% women, mean age 46.9, mean BMI 34.9 kg/m^2^) who responded to acute treatments were re-randomized to placebo (*N* = 34) or naltrexone/bupropion (*N* = 32) for 16 weeks; 86.3% completed posttreatment assessments. Mixed models and generalized estimating equations comparing maintenance treatments (naltrexone/bupropion *v.* placebo) included main and interactive effects of acute treatments.

**Results:**

Intention-to-treat binge-eating remission rates following maintenance treatments were 50.0% (*N* = 17/34) for placebo and 68.8% (*N* = 22/32) for naltrexone/bupropion. Placebo following response to acute treatment with naltrexone/bupropion was associated with significantly decreased probability of binge-eating remission, increased binge-eating frequency, and no weight loss. Naltrexone/bupropion following response to acute treatment with naltrexone/bupropion was associated with good maintenance of binge-eating remission, low binge-eating frequency, and significant additional weight loss.

**Conclusions:**

Adult patients with BED with co-occurring obesity who have good responses to acute treatment with naltrexone/bupropion should be offered maintenance treatment with naltrexone/bupropion.

## Introduction

Binge-eating disorder (BED) is a prevalent (Kessler et al., 2013; Udo & Grilo, 2018) and costly public health problem (Streatfeild et al., 2021). BED is associated with elevated rates of psychiatric/medical disorders and psychosocial impairments (Udo & Grilo, 2018; Udo & Grilo, 2019) and predicts future medical conditions (Hudson et al., 2010). BED is defined by recurrent binge eating episodes (subjective loss-of-control while consuming unusually large quantities of food), marked distress, and the absence of inappropriate weight-compensatory behaviors (American Psychiatric Association [APA], 2013). While BED is associated strongly with obesity (Kessler et al., 2013; Udo & Grilo, 2018), this psychiatric disorder has distinct behavioral, psychological, and neurobiological features than the medical diagnosis of obesity (Boswell, Potenza, & Grilo, 2021).

Despite the high morbidity associated with BED, it remains underrecognized, and most people with BED go untreated (Coffino, Udo, & Grilo, 2019). Recent controlled treatment research has identified certain psychological (Grilo, 2017) and pharmacological (McElroy, 2017) treatments with acute efficacy for BED, although most fail to produce weight loss (Hilbert et al., 2019; Reas & Grilo, 2021). The strong association between BED and obesity (Kessler et al., 2013; Udo & Grilo, 2018) coupled with associated medical concerns, including elevated cardiometabolic risks *and* patients’ preferences for treatment goals – which frequently include weight loss – indicate that focusing solely on binge eating or weight is a false dichotomy (Cardel et al., 2022). Growing evidence suggests that behavioral weight loss (BWL), a ‘generalist’ and disseminable behavioral treatment, results in acute outcomes in BED that approximate those with ‘specialist’ psychological treatments (such as cognitive behavioral therapy) but with the advantage of associated weight losses (Grilo, Masheb, Wilson, Gueorguieva, & White, 2011; Grilo, White, Masheb, Ivezaj, Morgan, & Gueorguieva, 2020b; Wilson, Wilfley, Agras, & Bryson, 2010).

In contrast to the growing evidence base for treatments with acute efficacy for BED, there is a dearth of controlled research examining maintenance treatments for responders to initial interventions. This gap in the literature appears particularly critical for pharmacological approaches for BED, which – in contrast to certain specific psychological treatments (Grilo et al., 2011; Wilson et al., 2010) – appear to be associated with rapid relapse following discontinuation (Grilo, Crosby, Wilson, & Masheb, 2012a). Although the potential role of pharmacotherapies to maintain benefits and prevent relapse with longer-term treatments represents an important clinical question, strikingly little research has addressed this issue for any eating disorder. To date, only three controlled trials have tested pharmacotherapy *maintenance* treatments for eating disorders: one study with anorexia nervosa found no effects for fluoxetine continuation (Walsh et al., 2006), one study with bulimia nervosa found continued fluoxetine improved outcomes and prevented relapse for those who responded to acute fluoxetine treatment (Romano, Halmi, Sarker, Koke, & Lee, 2002), and one study with BED found that continued lisdexamfetamine reduced relapse risk relative to placebo in those who responded to acute lisdexamfetamine treatment (Hudson, McElroy, Ferreira-Cornwell, Radewonuk, & Gasior, 2017).

This report describes a prospective, randomized, double-blind, placebo-controlled test of the efficacy of naltrexone/bupropion maintenance treatment following successful acute treatments for BED comorbid with obesity. Naltrexone/bupropion combination is an FDA-approved medication for the treatment of obesity (Yanovski & Yanovski, 2015). The putative mechanisms of naltrexone/bupropion are relevant for both binge eating and obesity. Naltrexone/bupropion is thought to regulate food intake and weight based on leptin's mechanisms of action (Billes & Greenway, 2011). Leptin's excitatory effects on pro-opiomelanocortin (POMC) neurons in the hypothalamus melanocortin system produce broad appetite-suppressant effects (Cowley et al., 2001). While stimulated POMC signaling decreases food intake and increases energy expenditure, it is inhibited by endogenous feedback (Cowley et al., 2001); thus, the logic for this combination is for bupropion to stimulate POMC neurons and for naltrexone to block endogenous feedback that inhibits POMC activity (Billes & Greenway, 2011). Naltrexone/bupropion has demonstrated acute efficacy for weight loss in patients with obesity (Greenway et al., 2009, 2010) and significantly enhances BWL outcomes in patients with obesity (Wadden et al., 2011). A *post-hoc* analysis of data from controlled trials testing naltrexone/bupropion for obesity suggested that among participants who achieved ⩾ 5% weight losses by 16-weeks, naltrexone/bupropion was associated with greater proportion than placebo of having ⩾ 5% weight loss maintenance at 12-month timepoints (le Roux, Fils-Aime, Camacho, Gould, & Barakat, 2022). The present prospective controlled *maintenance* treatment study follows the recent *acute* treatment trial examining naltrexone/bupropion and BWL for BED comorbid with obesity (Grilo et al., 2022). Patients categorized as ‘responders’ (defined as 65% or greater reduction in binge-eating frequency) following acute 16-week treatments were re-randomized, in double-blind fashion, with the initial acute treatment as a stratifying variable, to either naltrexone/bupropion or placebo for 16-weeks to examine maintenance without any additional behavioral or psychotherapeutic interventions.

We hypothesized that participants receiving naltrexone/bupropion would maintain reductions in binge-eating frequency and weight loss attained with acute treatment while participants receiving placebo would show increases in binge-eating frequency and in weight. We hypothesized that initial acute treatment condition (naltrexone/bupropion or BWL therapy) would moderate these maintenance treatment outcomes.

## Method

### Procedure

This single-site RCT was approved by the Yale institutional review board and included a data safety and monitoring plan with a physician safety monitor. Participants provided written informed consent.

### Participants

Participants for this controlled maintenance trial were eligible if they participated in the initial acute trial and were categorized as ‘responders’ following initial acute 16-week treatments in a randomized double-blind study testing naltrexone/bupropion and BWL, alone and combined, for BED in persons with obesity. The initial acute trial, which was reported previously (Grilo et al., 2022), used a 2 × 2 balanced factorial design with the following four conditions: placebo (*N* = 34), naltrexone/bupropion (*N* = 32), BWL plus placebo (*N* = 35), or BWL plus naltrexone/bupropion (*N* = 35). When patients initially enrolled in the treatment trial, they were informed and they consented to two treatment stages –i.e. the acute treatment stage (Grilo et al., 2022) and a second stage that would test pharmacotherapy maintenance if they responded to the initial treatment. Accordingly, participation in this maintenance treatment study was anticipated when participants consented to the 2-stage (initial acute ‘Stage 1’ plus maintenance ‘Stage 2’) treatments without an opt-out (i.e. if they responded to Stage 1, they participated in Stage 2, unless medically contraindicated).

Eligibility for the initial acute trial required *DSM-5* (APA, 2013) criteria for BED, ages 18–70 years old, and a body mass index (BMI; kg/m^2^) ⩾30.0 and ⩽50.0 (or ⩾27.0 with obesity-related comorbidity). The initial trial employed minimal exclusion criteria (to enhance generalizability) comprising clinical issues that, regardless of clinical setting, would require alternative treatments or represent contraindications to naltrexone/bupropion. Exclusionary criteria for the initial acute trial and for the current maintenance trial included: concurrent treatment for eating/weight disorders, taking contraindicated medications (e.g. opiates), uncontrolled medical conditions or contraindications to naltrexone/bupropion (e.g. seizure history, bulimia nervosa or anorexia nervosa history, cardiovascular disease, psychosis/bipolar disorder, systolic blood pressure > 160 mmHg, diastolic blood pressure > 100 mmHg, or heart rate > 100 beats/min), and pregnancy/breastfeeding.

Successful treatment ‘response’ to the initial acute 16-week treatments was defined as 65% reduction (relative to baseline) in past-month frequency of binge-eating at posttreatment. The 65% reduction threshold as the definition for ‘response’ to treatment was previously used in an adaptive stepped-care treatment trial to determine whether to continue treatment (for ‘responders’) or to switch to an alternative treatment in the case of ‘non-responders’ (Grilo et al., 2020b). The 65% definition was adopted, in part, because several studies reported that this cut-point, originally defined empirically using signal detection methods, reliably predicted treatment outcomes for BED with pharmacotherapy (Grilo, Masheb, & Wilson, 2006) and reductions in both binge-eating and weight through 12-month follow-ups with BWL (Grilo, White, Wilson, Gueorguieva, & Masheb, 2012b).

Participants meeting or exceeding this 65% or greater reduction threshold were categorized as ‘responders’ and included in the current maintenance treatment RCT. Binge-eating frequency (for the previous month – i.e. 28 calendar days) was assessed using the Eating Disorder Examination Interview (EDE; Fairburn, Cooper, and O'Connor, 2008) as part of the comprehensive posttreatment evaluation (see Grilo et al., 2022). The EDE was administered by doctoral-level assessors who were blinded to treatment conditions. The posttreatment evaluation was performed immediately following completion of the initial acute treatments and eligible participants were randomized and began this maintenance treatment study that same week.

The 66 participants had mean age of 46.92 (s.d. = 12.15) years and mean BMI of 34.93 (s.d. = 5.14) kg/m^2^; 84.8% (*N* = 56) were female, 87.9% (*N* = 58) attended/finished college, and 71.2% (*N* = 47) were White. Table 1 summarizes the participants' sociodemographic characteristics as well the specific Stage 1 treatments (2 × 2 balanced factorial design) received for the Stage 2 RCT participants, overall (*N* = 66) and separately for the placebo (*N* = 34) and naltrexone/bupropion (*N* = 32) treatment conditions.

**Table 1.** Sociodemographic characteristics overall and by treatment condition

	Overall		Placebo		Naltrexone/Bupropion			
	*N* = 66		*N* = 34		*N* = 32		Test Statistic	*p* value
Age, *N*, *M* (s.d.)	49.9	(12.1)	43.6	(11.8)	(50.4)	11.7	5.43	0.02
Sex (*N*, %)							1.94	0.38
Female	56	84.8%	28	82.4%	28	87.5%		
Male	9	13.6%	6	17.6%	3	9.4%		
Other	1	1.5%	0	0.0%	1	3.1%		
Race (*N*, %)							2.93	0.40
Asian	4	6.1%	3	8.8%	1	3.1%		
Black	10	15.2%	5	14.7%	5	15.6%		
Multiracial	5	7.6%	4	11.8%	1	3.1%		
White	47	71.2%	22	64.7%	25	78.1%		
Ethnicity (*N*, %)							0.96	0.33
Hispanic or Latinx	9	13.6%	6	17.6%	3	9.4%		
Not Hispanic or Latinx	57	86.4%	28	82.4%	29	90.6%		
Sexual Orientation (*N*, %)							2.96	0.23
Bisexual	2	3.0%	2	5.9%	0	0.0%		
Gay or Lesbian	0	0.0%	0	0.0%	0	0.0%		
Heterosexual	63	95.5%	32	94.1%	31	96.9%		
Other	1	1.5%	0	0.0%	1	3.1%		
Education, (*N*, %)							2.87	0.41
High School	8	12.1%	2	5.9%	6	18.8%		
Some college	20	30.3%	11	32.4%	9	28.1%		
College degree	16	24.2%	8	23.5%	8	25.0%		
More than College	22	33.3%	13	38.2%	9	28.1%		
Stage 1 Acute Treatment								
Placebo	13	19.7%	7	20.6%	6	18.8%		
Naltrexone/Bupropion	12	18.2%	7	20.6%	5	15.6%		
BWL + Placebo	18	27.3%	9	26.5%	9	28.1%		
BWL + Naltrexone/Bupropion	23	34.8%	11	32.4%	12	37.5%		

*Note*: Test statistic = χ^2^ for categorical variables and ANOVAs for dimensional variables comparing the treatments.

*p* values are for 2-tailed tests. *M*, mean; s.d., standard deviation; *N*, number. Sex, race, and ethnicity were self-reported.

Stage 1 Acute Treatment variable show the distribution of the four conditions received prior to this Stage 2 maintenance trial.

### Assessments

Assessment procedures were performed by trained/monitored doctoral-level research-clinicians who were independent from and blinded to treatments. The *Eating Disorder Examination* Interview (EDE; 16th-edition; Fairburn et al., 2008) was administered to assess binge-eating frequency and eating-disorder psychopathology at baseline and post-treatment. The EDE is often used as a primary measure in treatment studies with eating-disorder RCTs and has demonstrated good inter-rater and test-retest reliability in studies with BED (Grilo et al., 2022; Grilo, Masheb, Lozano-Blanco, & Barry, 2004). *Weight and height* were measured at baseline and weight was measured at week two, monthly, and at post-treatment. When being weighed using digital scales, participants were instructed to wear lightweight clothing and remove their shoes.

### Randomization and blinding procedures

The randomization schedule, developed by a biostatistician, assigned participants categorized as ‘responders’ to either naltrexone/bupropion or placebo (double-blind) for 16-weeks using stratified blocked randomization with the initial acute treatment as a stratifying variable. The schedule comprised random block sizes of two and four to obviate secular trends and to yield approximately equal proportions. Participants and clinicians remained blinded as to whether they had received naltrexone/bupropion in their initial acute treatment stage, in addition to once again being blinded to their randomized medication condition in this trial. Assessors of outcomes were blinded to whether participants had received BWL during their prior Stage 1 treatment in addition to the (double-blind) medication in both the prior and current trials.

### Treatments

Treatment involved solely the double-blind medication (naltrexone/bupropion or placebo). There were no additional behavioral or psychotherapeutic interventions during this maintenance trial. Naltrexone/bupropion combination full dosing comprised naltrexone-sustained-release (32 mg/day) plus bupropion-sustained-release (360 mg/day) as in previous trials with obesity (Greenway et al., 2009, 2010; Wadden et al., 2011). Two capsules, each containing 8 mg naltrexone and 90 mg bupropion, were taken twice daily. Placebo was taken in capsules matched in appearance and frequency (i.e. two capsules twice daily).

Our re-randomization pharmacotherapy protocol took both the acute and the maintenance pharmacotherapy assignments into consideration to maximize participant safety and reduce potential confounds attributable to unblinding. We provide the four re-randomization schedules in online Supplemental Material Text and Supplemental Figure. Briefly, participants switching from naltrexone/bupropion to placebo had a down-titration of naltrexone bupropion as part of their double-blind schedule, as well as up-titration of placebo. Participants switching from placebo to naltrexone/bupropion had an up-titration following procedures in previous trials with obesity (Greenway et al., 2009, 2010; Wadden et al., 2011) and in the acute trial with BED (Grilo et al., 2022). Participants staying on naltrexone/bupropion neither down-titrated nor up-titrated. To preserve the blind, and because there were no safety concerns related to inactive medication, participants staying on placebo paralleled those who were switching from placebo to naltrexone/bupropion.

At the first (initial) maintenance trial study visit, study physicians delivered the pharmacotherapy, which focused on medication management (compliance, safety, and side-effects). Additional psychotherapeutic or behavioral interventions were proscribed. Monthly medication refills were accompanied by re-reviewing medication compliance and dosing schedules; pill bottles were returned for pill counts at post-treatment. Side-effect and safety checklists were performed monthly by research clinicians.

### Statistical analysis

Sample size for this maintenance trial was calculated for the comparison between naltrexone/bupropion and placebo, based on estimated rates of response to acute treatments, considering clinically meaningful effects sizes, and performing sensitivity analyses for different outcomes. Power analyses could not be based directly on data from RCTs testing acute effects of naltrexone/bupropion for obesity (Greenway et al., 2009, 2010); however, since those RCTs reported 12-month outcome data with effects sizes in the medium-to-large ranges, this seemed supportive of our approach. Based on 44 randomized participants per treatment and allowing for 20% dropout (that is, assuming 35–36 completers in each treatment group), we estimated at least 80% power to detect a medium to large effect size (*d* = 0.68) for the difference between naltrexone/bupropion *v.* placebo at 2-tailed alpha of 0.05. This was a conservative calculation as mixed models allow us to use all available data on individuals, not just data on completers. For remission rates, with 36 individuals per treatment group we estimated ability to detect the following clinically meaningful differences in proportions (15% *v.* 45%, 30% *v.* 63%, 45% *v.* 77%) with 80% power at 2-tailed alpha of 0.05. Our intent-to-treat sample (66 total) ended up only slightly lower than the target (70 total) and thus we were well-powered for the a priori specified clinically meaningful effects.

Analyses to compare treatments were all intention-to-treat and were performed for all randomized patients who attended the first treatment session. The two co-primary outcome variables were binge eating and weight loss. Binge eating was analyzed in two complementary ways – i.e. as a continuous variable (monthly frequency of episodes) and as a categorical variable. The categorical outcome variable was binge-eating remission, defined as zero episodes during the previous 28 days on the Eating Disorder Examination interview at posttreatment; any missing data were imputed as failure (i.e. non-remission). Weight (measured) was analyzed as a continuous outcome (percent weight-loss from the beginning of the maintenance trial) and as a categorical outcome. The categorical outcome was clinically-significantly weight loss, defined as 5% or greater weight loss (Brown, Buscemi, Milsom, Malcolm, & O'Neil, 2016; Wing et al., 2011) between the maintenance trial baseline and posttreatment assessments; missing data were imputed as failure.

For analyses of continuous variables, intention-to-treat analyses used all available data in mixed models without imputation. Variables not conforming to normality were log-transformed prior to analysis. Mixed effects models were fitted with fixed factors including medication during this maintenance trial (naltrexone/bupropion *v.* placebo), medication during initial acute treatment (naltrexone/bupropion *v.* placebo), BWL therapy during initial acute treatment (yes, no), time (all relevant time points), and all possible interactions. In each model, we considered different error structures and selected the best-fitting structure using the Schwarz's Bayesian Criterion. Statistical testing was performed at 0.05 significance level.

For categorical variable outcomes, intention-to-treat analyses involved generalized estimating equations (GEE) models. Logit link function was used with binomial response distribution. Predictor variables were the same as in the mixed models above. If models encountered convergence problems with all possible interactions, interactions were dropped, and the model refitted until a model with no convergence issues was identified.

We explored whether time of measurement (before or during the COVID pandemic) affected the results by including an indicator for timing of measurement as a covariate. The results did not change substantively and therefore the final models are not adjusted for COVID.

## Results

### Randomization and participant flow through treatment study

**Figure 1.**
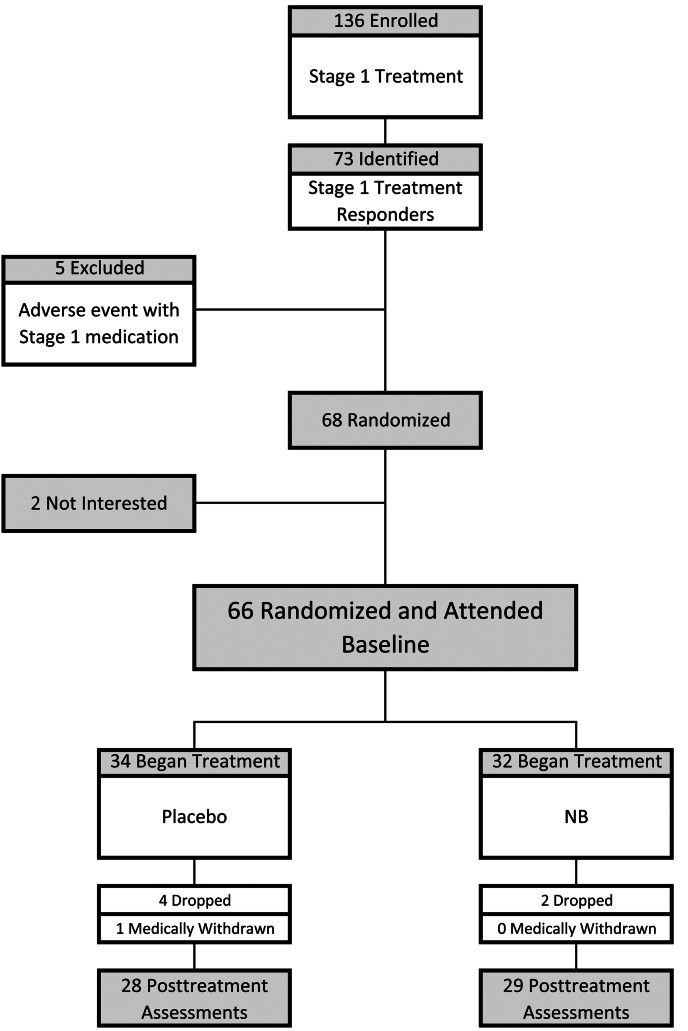
Participant flow throughout the study. Participant flow through this randomized double-blind controlled trial (RCT) testing naltrexone/bupropion (NB) *v.* placebo for continued treatment of patients with binge-eating disorder who responded successfully to acute treatments (Stage 1). Stage 1 treatment (*N* = 136) was a RCT testing naltrexone/bupropion and behavioral weight loss (BWL), alone and together, using a 2 × 2 balanced factorial design. Table 1 shows the Stage 1 treatments (a stratifying variable in the randomization) received by the participants in this Stage 2 maintenance RCT. Of the 136 participants receiving Stage 1 treatments, 73 were categorized as treatment responders (of those, 5 were excluded due to adverse events during acute Stage 1 treatments), and 66 were randomized and attended baseline treatment session for this RCT. Of the 66 participants, 59 (89.4%) completed treatments, and 57 86.3% completed posttreatment assessments. Of the *N* = 66 participants in this Stage 2 maintenance RCT, during the Stage 1 (initial acute) treatment trial, 35 (53.0%) received naltrexone/bupropion and 41 (62.1%) received behavioral weight loss therapy. Table 1 summarizes the specific Stage 1 treatments (2 × 2 balanced factorial design) received by the Stage 2 RCT participants, overall (*N* = 66) and separately for the placebo (*N* = 34) and naltrexone/bupropion (*N* = 32) treatment conditions.

Figure 1 (CONSORT) summarizes participant flow throughout the study. Of the 136 participants who began acute treatments (28), 73 were categorized as treatment responders (of these, five had experienced adverse events while on acute pharmacotherapy and were therefore excluded from the maintenance treatment trial), and 66 were randomized and attended baseline session of this maintenance trial. Of the 66 participants, *N* = 34 received placebo and *N* = 32 received naltrexone/bupropion. Overall, 59 of the 66 (89.4%) completed the maintenance treatments. Medication was tolerated well, with only *N* = 1 participant withdrawn due to a medical concern (from placebo condition), As summarized in the online Supplemental Table S1, the medication was not associated with serious harms and the patterning of reported side effects is generally similar to that reported for the pivotal trials for obesity (Greenway et al., 2010). Post-treatment assessments were obtained for 86.4% (*N* = 57/66) of participants.

### Primary outcomes

Table 2 shows descriptive statistics for primary outcomes (binge eating and weight loss) and Table 3 shows the test statistics and *p* values for all the main and interaction effects in the statistical models for the outcomes. Online Supplemental Table S2 shows descriptive statistics for secondary outcomes.

**Table 2.** Clinical measures by treatment conditions

	Placebo			Naltrexone/Bupropion		
	*N* = 34			*N* = 32		
	*n*	*M*	s.d.	*n*	*M*	s.d.
Binge-Eating Frequency (episodes in past month)						
Pre-Treatment	34	0.74	1.50	32	1.13	2.17
Post-Treatment	28	3.25	7.06	29	0.86	2.15
*Change*	28	2.82	7.22	29	0.00	0.96
Weight (pounds)						
Pre-Treatment	34	223.34	46.49	32	207.35	35.42
Post-Treatment	26	226.16	48.21	29	202.32	41.82
*Change*	26	2.42	8.44	29	−5.88	9.65
*% Change*	26	0.01	0.04	29	−0.03	0.05
BMI (kg/m^2^)						
Pre-Treatment	34	35.40	5.21	32	34.43	5.10
Post-Treatment	26	36.07	5.90	29	33.52	5.99
*Change*	26	0.39	1.34	29	−1.04	1.70

*Note*: Binge-eating frequency was monthly, defined as number of episodes during the previous 28 days, based on the Eating Disorder Examination Interview; BMI, body mass index; *N*, number; M, mean; s.d., standard deviation.

**Table 3.** Analytic model findings for primary outcomes.

	Binge-Eating Frequency (EDE Interview)	Binge-Eating Remission (EDE Interview, 0 Episodes)	Percent Weight Loss
BWL _Stage1_	*F*_(1, 54.9)_ = 0.16, *p* = 0.69	χ^2^(1) = 0.00, *p* = 0.99	*F*_(1, 58.1)_ = 2.63, *p* = 0.11
MED _Stage1_	*F*_(1, 54.9)_ = 0.00, *p* = 0.97	χ^2^(1) = 0.55, *p* = 0.46	*F*_(1, 58.1)_ = 1.84, *p* = 0.18
BWL _Stage1_*MED _Stage1_	*F*_(1, 54.9)_ = 0.23, *p* = 0.63	χ^2^(1) = 0.73, *p* = 0.39	*F*_(1, 58.1)_ = 0.00, *p* = 0.96
MED _Stage2_	*F*_(1, 54.9)_ = 0.28, *p* = 0.60	χ^2^(1) = 0.12, *p* = 0.73	***F***_(_**_1, 58.1)_ = 11.09, *p* = 0.001**
BWL _Stage1_*MED _Stage2_	*F*_(1, 54.9)_ = 0.28, *p* = 0.60	χ^2^(1) = 0.13, *p* = 0.72	*F*_(1, 58.1)_ = 1.23, *p* = 0.27
MED _Stage1_*MED _Stage2_	*F*_(1, 54.9)_ = 0.13, *p* = 0.72	χ^2^(1) = 0.47, *p* = 0.50	*F*_(1, 58.1)_ = 0.32, *p* = 0.58
BWL _Stage1_*MED _Stage1_*MED_Stage2_	*F*_(1, 54.9)_ = 0.34, *p* = 0.56	χ^2^(1) = 0.10, *p* = 0.75	*F*_(1, 58.1)_ = 0.85, *p* = 0.36
Time	*F*_(1, 51)_ = 0.79, *p* = 0.38	χ^2^(1) = 0.35, *p* = 0.55	*F*_(3, 137)_ = 2.37, *p* = 0.07
BWL _Stage1_*Time	*F*_(1, 51)_ = 1.70, *p* = 0.20	χ^2^(1) = 1.96, *p* = 0.16	*F*_(3, 137)_ = 0.80, *p* = 0.50
MED _Stage1_*Time	***F***_(1, 51)_ **= 6.21, *p* = 0.02**	χ^2^**(1) = 6.28, *p* = 0.01**	*F*_(3, 137)_ = 0.93, *p* = 0.43
BWL _Stage1_*MED _Stage1_*Time	*F*_(1, 51)_ = 0.93, *p* = 0.34	χ^2^(1) = 1.11, *p* = 0.29	*F*_(3, 137)_ = 0.54, *p* = 0.65
MED _Stage2_*Time	*F*_(1, 51)_ = 3.52, *p* = 0.07	χ^2^(1) = 1.40, *p* = 0.24	***F***_(3, 137)_ **= 4.16, *p* = 0.01**
BWL _Stage1_*MED _Stage2_*Time	*F*_(1, 51)_ = 0.76, *p* = 0.39	χ^2^(1) = 0.00, *p* = 0.95	*F*_(3, 137)_ = 1.34, *p* = 0.27
MED _Stage1_*MED _Stage2_*Time	***F***_(_**_1, 51)_ = 6.31, *p* = 0.02**	χ^2^**(1) = 3.81, *p* = 0.05**	*F*_(3, 137)_ = 0.14, *p* = 0.94
BWL_Stage1_*MED_Stage1_*MED_Stage2_*Time	*F*_(1, 51)_ = 1.24, *p* = 0.27	–	*F*_(3, 137)_ = 0.61, *p* = 0.61

Note: All analyses were intention-to-treat (with *N* = 66). Mixed models were used for continuous variables (binge-eating frequency and percent weight loss) and generalized estimating equations (GEE) for categorical variables (binge-eating remission); the final interaction term was not included in the GEE model as the model with this interaction failed to converge.

**BWL**, Behavioral Weight Loss; **MED**, naltrexone/bupropion *v.* placebo; **Time**, assessment timepoints; *, interaction (e.g. BWL*Time denotes BWL-by-Time interaction effect in statistical model; **Stage 1**, acute intervention. **Stage 2**, maintenance treatment. **EDE**, Eating Disorder Examination Interview administered at pre-treatment and post-treatment. Remission was defined as zero binge-eating episodes during past month. Weight was measured monthly; percent weight loss was calculated relative to Stage 2 maintenance pre-treatment weight.

#### Binge-eating remission

**Figure 2.**
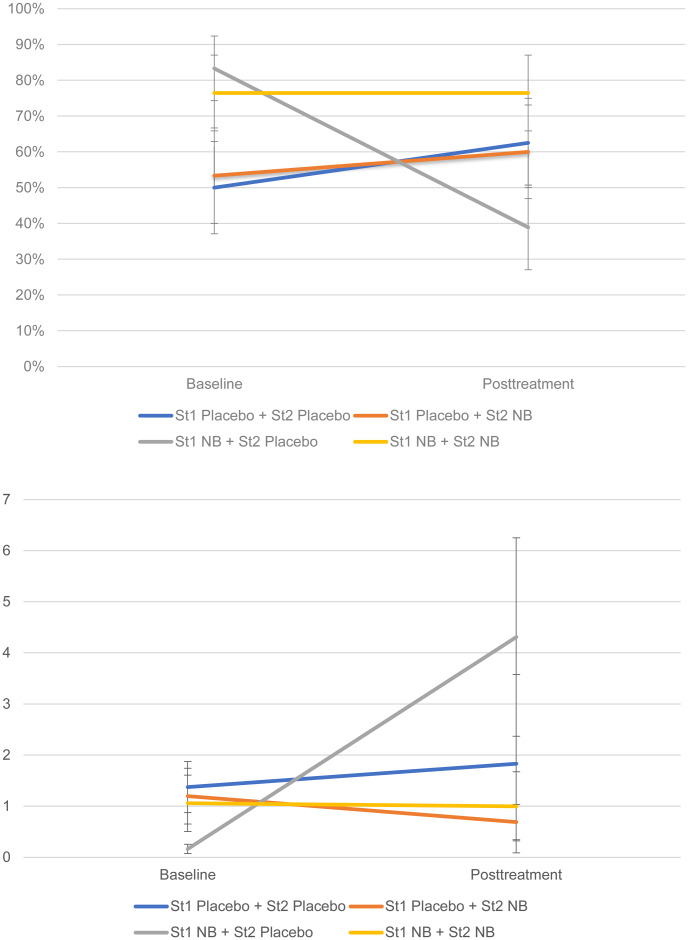
Binge-eating across the treatment medication conditions. (a) (top panel) caption. Binge-eating remission rates during the maintenance treatment (Stage 2) at baseline and at post-treatment illustrating the significant interaction effects between treatment medication in the acute (Stage 1) and maintenance (Stage 2) phases. Remission rates are defined as zero episodes of binge eating during the last 28 days assessed using the Eating Disorder Examination Interview. The rates are based on the intention-to-treat sample (*N* = 66) with any missing data imputed as failure to remit. The four lines show the raw rates (error bars indicate standard errors) of binge-eating remission separately for naltrexone/bupropion (NB) and placebo conditions during maintenance (Stage 2) treatment separately by naltrexone/bupropion or placebo during initial acute (Stage 1) treatments. *N* = 17 received Stage 1 NB + Stage 2 NB; *N* = 18 received Stage 1 NB + Stage 2 Placebo; *N* = 15 received Stage 1 Placebo + Stage 2 NB; and *N* = 16 received Stage 1 Placebo + Stage 2 Placebo. St1 = Stage 1 (acute treatment); St2 = Stage 2 (maintenance treatment); NB, naltrexone/bupropion. (b) (bottom panel) caption. Frequency of binge eating episodes during the past 28 days for the maintenance treatment (Stage 2) at baseline and at post-treatment (assessed using the Eating Disorder Examination interview). This Figure illustrates the significant interaction effects between treatment medication in the acute (Stage 1) and maintenance (Stage 2) phases. The four lines show the raw frequencies (error bars indicate standard errors) of binge-eating episodes separately for naltrexone/bupropion and placebo conditions during maintenance (Stage 2) treatment separately by naltrexone/bupropion or placebo during initial acute (Stage 1) treatments. *N* = 17 received Stage 1 NB + Stage 2 NB; *N* = 18 received Stage 1 NB + Stage 2 Placebo; *N* = 15 received Stage 1 Placebo + Stage 2 NB; and *N* = 16 received Stage 1 Placebo + Stage 2 Placebo. St1, Stage 1 (acute treatment); St2, Stage 2 (maintenance treatment); NB, naltrexone/bupropion

Intention-to-treat remission rates following the 16-week maintenance treatments were 50.0% (*N* = 17/34) for placebo and 68.8% (*N* = 22/32) for naltrexone/bupropion maintenance treatment; this overall difference was not significant (Fisher's exact test *p* = 0.14). However, GEE models (Table 3) evaluating time effects and interactive effects between acute treatments (Stage 1) and maintenance treatments (Stage 2) revealed a borderline significant interaction between medication during acute treatment, medication during maintenance treatment and time (χ^2^(1) = 3.81, *p* = 0.05) and a significant interaction between medication during acute treatment and time during maintenance treatment (χ^2^(1) = 6.28, *p* = 0.01). The estimated probability for remission decreased among those who had naltrexone/bupropion during acute treatment but received placebo during maintenance treatment (from 0.83, s.e. = 0.09 at the end of active treatment to 0.45, s.e. = 0.13 at end of maintenance treatment) whereas it stayed relatively unchanged for those who received naltrexone/bupropion during both the acute and maintenance treatments phases (from 0.74, s.e. = 0.12 to 0.80, s.e. = 0.10). For those who did not have naltrexone/bupropion during acute treatment, the estimated probability of remission increased regardless of treatment in the maintenance phase (from 0.51, s.e. = 0.09 at the end of active treatment to 0.76, s.e. = 0.08 at the end of maintenance treatment). Figure 2a illustrates the significant interaction effects between treatment medication in the acute (Stage 1) and maintenance (Stage 2) treatments.

#### Binge-eating frequency

Mixed models analyses of binge-eating frequency (episodes during the past month based on the Eating Disorder Examination interview) revealed a significant interaction between medication during acute treatment, medication during maintenance treatment and time (*F*_(1,51)_ = 6.31, *p* = 0.02) and a significant interaction between medication during acute treatment and time during maintenance treatment (*F*_(1,51)_ = 6.21, *p* = 0.02) (Table 3). Binge-eating frequency did not change significantly (i.e. remained low) in participants who received naltrexone/bupropion during both acute and maintenance treatments but increased significantly in those who received placebo during maintenance treatment after having received naltrexone/bupropion during initial acute treatment. At the end of stage 2 treatment there was a significant difference between the active and placebo groups among those on active treatment in stage 1 (*F*_(1,95.6)_ = 5.32, *p* = 0.02). Binge-eating frequency did not significantly differ by maintenance treatment in participants who did not receive active medication treatment in stage 1. Figure 2b illustrates the significant interaction effects between treatment medication treatment in the acute (Stage 1) and maintenance (Stage 2) treatments.

#### Percent weight loss

**Figure 3.**
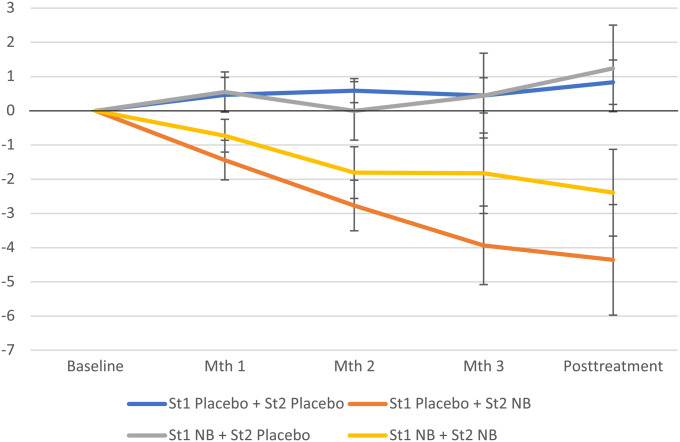
Percent weight loss across the treatment medication conditions. Percent weight loss (from baseline start of maintenance treatment) calculated using measured values at baseline, measured monthly during maintenance treatment, and measured at post-treatment. Figure illustrates the significant interaction effects between treatment medication in the acute (Stage 1) and maintenance (Stage 2) phases. The four lines show the raw means (error bars indicate standard errors) for naltrexone/bupropion and placebo conditions during maintenance (Stage 2) treatment separately by naltrexone/bupropion or placebo during initial acute (Stage 1) treatments. *N* = 17 received Stage 1 NB + Stage 2 NB; *N* = 18 received Stage 1 NB + Stage 2 Placebo; *N* = 15 received Stage 1 Placebo + Stage 2 NB; and *N* = 16 received Stage 1 Placebo + Stage 2 Placebo. St1, Stage 1 (acute treatment); St2, Stage 2 (maintenance treatment); Mth, Month; NB, naltrexone/bupropion.

Table 2 shows weight values at baseline and post-treatment and changes. Mixed models of percent weight loss measured monthly and at posttreatment revealed a significant interaction between medication maintenance treatment and time (*F*_(3137)_ = 4.16, *p* = 0.008) and a significant main effect of stage 2 treatment (*F*_(1,58.1)_ = 11.09, *p* = 0.002) (Table 3). There was significant weight loss in the group that received naltrexone/bupropion during maintenance treatment (*F*_(3136)_ = 5.95, *p* = 0.0008) but not in the group that received placebo during maintenance treatment (*F*_(3137)_ = 0.44, *p* = 0.73); naltrexone/bupropion and placebo differed significantly (*p*s < 0.001) at all monthly time points (except month 1) during this maintenance treatment. Figure 3 summarizes percent weight loss throughout the course of the maintenance treatments shown separately for those who received naltrexone/bupropion or placebo during the acute (Stage 1) treatments.

#### Attaining 5% weight loss

The group receiving naltrexone/bupropion maintenance treatment was significantly more likely than the group receiving placebo to attain ⩾5% weight loss during the maintenance period (22% *v.* 3%; Fisher's exact test value = 0.02).

## Discussion

The present prospective controlled *maintenance* treatment study of adults with BED and obesity, only the second maintenance treatment study of any kind performed to date with BED, tested the effectiveness of naltrexone/bupropion for maintenance following successful acute treatments for BED comorbid with obesity. Participants who had good outcomes during a controlled *acute* treatment trial testing naltrexone/bupropion and BWL treatments for BED comorbid with obesity (Grilo et al., 2022) were re-randomized, in double-blind fashion, to either naltrexone/bupropion or to placebo for 16 weeks. Findings for both binge-eating and weight loss outcomes (considered both continuously and categorically) converged to indicate strongly that patients with good responses to acute treatment with naltrexone/bupropion should be offered maintenance treatment with naltrexone/bupropion.

Specifically, maintenance treatment with placebo following good response to acute treatment with naltrexone/bupropion was associated with significantly increased binge-eating frequency, significantly decreased probability of binge-eating remission, and no weight loss. In sharp contrast, maintenance treatment with naltrexone/bupropion following good response to acute treatment with naltrexone/bupropion was associated with good maintenance of binge-eating remission and low frequency of binge-eating frequency and significant *further* weight loss. Naltrexone/bupropion resulted in significantly greater likelihood than placebo of attaining 5% or greater weight loss during the maintenance period (22% *v.* 3%); moreover, amongst those who received naltrexone/bupropion during both treatment stages, weight losses during the acute (mean of 5% loss) and maintenance (mean of 3% additional loss) culminated in over 8% weight losses. Research on patients with obesity (albeit without consideration of BED) has found that such modest weight losses are associated with important health benefits such as significantly improved glycemic control and reductions in cardiometabolic abnormalities (Brown et al., 2016; Wing et al., 2011).

Our findings supporting naltrexone/bupropion maintenance treatment parallel those reported by Hudson et al. (2017), in their randomized double-blind placebo-controlled maintenance study, in which initial responders to open-label active lisdexamfetamine were subsequently randomly withdrawn in double-blind double-controlled manner. That trial, however, which focused on binge-eating relapse, did not consider weight loss outcomes. More broadly, our findings regarding the worsening outcomes associated with the placebo maintenance condition amongst those treated initially with naltrexone/bupropion are consistent with findings that relapse is both frequent and significantly more common following completion of acute fluoxetine treatment than cognitive-behavioral therapy in BED (Grilo et al., 2012a).

This controlled pharmacological maintenance study also provided new information about maintenance treatment following BWL treatment for BED. Maintenance medication (naltrexone/bupropion *v.* placebo) did not appear to be associated with any significant changes in the courses of binge-eating and weight changes in those who responded to initial acute treatments with BWL therapy. Such findings provide further support for the potential durability of effectiveness of BWL therapy for BED given that re-randomization to placebo medication for the maintenance period was not associated with significant changes (i.e. no significant interaction effects, which suggested that the initial acute improvements were maintained). These experimental findings extend prior naturalistic prospective follow-up studies of BWL therapy indicating good maintenance after completing initial acute treatment through 12 months (Grilo et al., 2011, 2020a; Wilson et al., 2010).

We note potential methodological strengths and limitations as context for interpreting the implications of these findings. Study strengths include the randomized double-blind design to test pharmacological maintenance treatment, pharmacotherapy delivered by trained/monitored faculty-level study physicians, independent assessments using well-validated measures, minimal exclusionary criteria intended to enhance generalizability, and high retention rates. A further methodological consideration concerns the nature of the initial acute treatment. In contrast to the *only* other experimental test of pharmacological maintenance in BED (Hudson et al., 2017), our experimental study of maintenance followed acute treatments comprising double-blind naltrexone/bupropion and/or BWL therapy in which initial responders were re-randomized to double-blind naltrexone/bupropion or placebo. Our re-randomization protocol was double-blind and accounted for treatment randomization assignments in both stage 1 and stage 2. Importantly, this complex design allowed for testing pharmacological maintenance effects following pharmacological or BWL treatments as our statistical models testing maintenance included acute treatments as both main and interaction terms. The sample size had limited power to detect smaller magnitude main or interaction effects of treatments.

We note that the generalizability of the findings to different clinical settings or to persons with different sociodemographic is uncertain. The participant group lacked robust diversity: 85% were female, 71% White, 95% heterosexual, and 88% attended at least some college; notably, however, the sample comprised some degree of racial/ethnic representation (15% Black and 14% Hispanic). White women comprise the ‘overwhelming’ majority of participants in treatment studies for eating disorders (Burnette, Luzier, Weisenmuller, & Boutte, 2022) and epidemiological studies have documented substantial disparities in help-seeking by people with BED, notably that men and people of color seek treatments at very low rates (Coffino et al., 2019). Collectively, these findings highlight the pressing need for treatment research studies to more aggressively recruit and effectively enroll more diverse participant groups and to investigate specific needs of different minority and cultural groups.

With the caution regarding generalizability noted with the broader context of the pressing need for greater diversity in treatment research for BED, we note several points for consideration. First, sex (Lydecker, Gueorguieva, Masheb, White, & Grilo, 2020) and race and ethnicity have not been found to moderate treatment outcomes for BED in aggregated analyses of psychosocial interventions (Thompson-Brenner et al., 2013) or of psychological, pharmacological, or multimodal interventions (Lydecker, Gueorguieva, Masheb, White, & Grilo, 2019). Importantly, Black participants have been found to have comparable or better binge-eating outcomes than White participants but were less likely to attain weight loss (Lydecker et al., 2019). Second, a RCT testing BWL and weight-loss medication performed at a community mental health center serving Hispanic/Latinx (Spanish-speaking-only) patients characterized by marked educational- and economic-disadvantages reported BED outcomes that approximate the RCT literature for BED with much more restrictive samples (Grilo & White, 2013). Such finding challenge, to some degree, concerns that evidence-based behavioral and pharmacological treatments cannot be effectively delivered to more diverse groups or in ‘real-world’ clinical settings. Third, the sociodemographic characteristics for the Stage 1 RCT (Grilo et al., 2022) and for those here in this Stage 2 RCT for responders were very similar. The Stage 1 RCT and Stage 2 RCT (present study) comprised (respectively): 84.8% *v.* 81.6% female; 71.2% *v.* 77.9% White, 15.2% *v.* 13.2% Black, 13.6% *v.* 14/7% Hispanic, and, in terms of education, 12.1% *v.* 15.4% high school, 30.3% *v.* 29.4% some college, 24.2% *v.* 23.5% college degree, and 33.3% *v.* 31.6% more than college. Thus, the sociodemographic composition of this non-responder study group in this maintenance RCT was very similar to that of the Stage 1 RCT thus suggesting the absence of selection bias or such confounds.

With these methodological considerations and potential limitations as context, we conclude that adult patients with BED with co-existing obesity who have good responses to acute treatment with naltrexone/bupropion should be offered maintenance treatment with naltrexone/bupropion. BWL for BED had durable outcomes regardless of pharmacological maintenance treatment.

## Supporting information

Grilo et al. supplementary material 1 Grilo et al. supplementary material

Grilo et al. supplementary material 2 Grilo et al. supplementary material

Grilo et al. supplementary material 3 Grilo et al. supplementary material

Grilo et al. supplementary material 4 Grilo et al. supplementary material

## Supplementary material

The supplementary material for this article can be found at https://doi.org/10.1017/S0033291723001800
