# Supplementary material for: Naltrexone plus bupropion combination medication maintenance treatment for binge-eating disorder following successful acute treatments: randomized double-blind placebo-controlled trial
Source: Psychol Med. 2023 Jun 27;53(16):7775–84. doi: 10.1017/S0033291723001800 (PMC10751383; doi:10.1017/S0033291723001800)
Supplement: Grilo et al. supplementary material 1 — Grilo et al. supplementary material [file S0033291723001800sup001.pdf]

Placebo (acute; Bottle A) → Placebo (maintenance; Bottle B)

Day 1<br>AM: 1 pill [ ]<br>*from Bottle A*<br>PM: 0 pills	Day 2<br>AM: 1 pill [ ]<br>*from Bottle A*<br>PM: 0 pills	Day 3<br>AM: 1 pill [ ]<br>*from Bottle A*<br>PM: 0 pills	Day 4<br>AM: 1 pill [ ]<br>*from Bottle A*<br>PM: 0 pills	Day 5<br>AM: 1 pill [ ]<br>*from Bottle A*<br>PM: 0 pills	Day 6<br>AM: 1 pill [ ]<br>*from Bottle A*<br>PM: 0 pills	Day 7<br>AM: 1 pill [ ]<br>*from Bottle A*<br>PM: 0 pills
Day 8<br>AM: 1 pill [ ]<br>*from Bottle B*<br>PM: 1 pill [ ]<br>*from Bottle B*	Day 9<br>AM: 1 pill [ ]<br>*from Bottle B*<br>PM: 1 pill [ ]<br>*from Bottle B*	Day 10<br>AM: 1 pill [ ]<br>*from Bottle B*<br>PM: 1 pill [ ]<br>*from Bottle B*	Day 11<br>AM: 1 pill [ ]<br>*from Bottle B*<br>PM: 1 pill [ ]<br>*from Bottle B*	Day 12<br>AM: 1 pill [ ]<br>*from Bottle B*<br>PM: 1 pill [ ]<br>*from Bottle B*	Day 13<br>AM: 1 pill [ ]<br>*from Bottle B*<br>PM: 1 pill [ ]<br>*from Bottle B*	Day 14<br>AM: 1 pill [ ]<br>*from Bottle B*<br>PM: 1 pill [ ]<br>*from Bottle B*
Day 15<br>AM: 2 pills [ ]<br>*from Bottle B*<br>PM: 1 pill [ ]<br>*from Bottle B*	Day 16<br>AM: 2 pills [ ]<br>*from Bottle B*<br>PM: 1 pill [ ]<br>*from Bottle B*	Day 17<br>AM: 2 pills [ ]<br>*from Bottle B*<br>PM: 1 pill [ ]<br>*from Bottle B*	Day 18<br>AM: 2 pills [ ]<br>*from Bottle B*<br>PM: 1 pill [ ]<br>*from Bottle B*	Day 19<br>AM: 2 pills [ ]<br>*from Bottle B*<br>PM: 1 pill [ ]<br>*from Bottle B*	Day 20<br>AM: 2 pills [ ]<br>*from Bottle B*<br>PM: 1 pill [ ]<br>*from Bottle B*	Day 21<br>AM: 2 pills [ ]<br>*from Bottle B*<br>PM: 1 pill [ ]<br>*from Bottle B*
Day 22<br>AM: 2 pills [ ]<br>*from Bottle B*<br>PM: 2 pills [ ]<br>*from Bottle B*	Day 23<br>AM: 2 pills [ ]<br>*from Bottle B*<br>PM: 2 pills [ ]<br>*from Bottle B*	Day 24<br>AM: 2 pills [ ]<br>*from Bottle B*<br>PM: 2 pills [ ]<br>*from Bottle B*	Day 25<br>AM: 2 pills [ ]<br>*from Bottle B*<br>PM: 2 pills [ ]<br>*from Bottle B*	Day 26<br>AM: 2 pills [ ]<br>*from Bottle B*<br>PM: 2 pills [ ]<br>*from Bottle B*	Day 27<br>AM: 2 pills [ ]<br>*from Bottle B*<br>PM: 2 pills [ ]<br>*from Bottle B*	Day 28<br>AM: 2 pills [ ]<br>*from Bottle B*<br>PM: 2 pills [ ]<br>*from Bottle B*

Placebo (acute) → NB (maintenance; Bottle A and B)

Day 1<br>AM: 1 pill [ ]<br>*from Bottle A*<br>PM: 0 pills	Day 2<br>AM: 1 pill [ ]<br>*from Bottle A*<br>PM: 0 pills	Day 3<br>AM: 1 pill [ ]<br>*from Bottle A*<br>PM: 0 pills	Day 4<br>AM: 1 pill [ ]<br>*from Bottle A*<br>PM: 0 pills	Day 5<br>AM: 1 pill [ ]<br>*from Bottle A*<br>PM: 0 pills	Day 6<br>AM: 1 pill [ ]<br>*from Bottle A*<br>PM: 0 pills	Day 7<br>AM: 1 pill [ ]<br>*from Bottle A*<br>PM: 0 pills
Day 8<br>AM: 1 pill [ ]<br>*from Bottle B*<br>PM: 1 pill [ ]<br>*from Bottle B*	Day 9<br>AM: 1 pill [ ]<br>*from Bottle B*<br>PM: 1 pill [ ]<br>*from Bottle B*	Day 10<br>AM: 1 pill [ ]<br>*from Bottle B*<br>PM: 1 pill [ ]<br>*from Bottle B*	Day 11<br>AM: 1 pill [ ]<br>*from Bottle B*<br>PM: 1 pill [ ]<br>*from Bottle B*	Day 12<br>AM: 1 pill [ ]<br>*from Bottle B*<br>PM: 1 pill [ ]<br>*from Bottle B*	Day 13<br>AM: 1 pill [ ]<br>*from Bottle B*<br>PM: 1 pill [ ]<br>*from Bottle B*	Day 14<br>AM: 1 pill [ ]<br>*from Bottle B*<br>PM: 1 pill [ ]<br>*from Bottle B*
Day 15<br>AM: 2 pills [ ]<br>*from Bottle B*<br>PM: 1 pill [ ]<br>*from Bottle B*	Day 16<br>AM: 2 pills [ ]<br>*from Bottle B*<br>PM: 1 pill [ ]<br>*from Bottle B*	Day 17<br>AM: 2 pills [ ]<br>*from Bottle B*<br>PM: 1 pill [ ]<br>*from Bottle B*	Day 18<br>AM: 2 pills [ ]<br>*from Bottle B*<br>PM: 1 pill [ ]<br>*from Bottle B*	Day 19<br>AM: 2 pills [ ]<br>*from Bottle B*<br>PM: 1 pill [ ]<br>*from Bottle B*	Day 20<br>AM: 2 pills [ ]<br>*from Bottle B*<br>PM: 1 pill [ ]<br>*from Bottle B*	Day 21<br>AM: 2 pills [ ]<br>*from Bottle B*<br>PM: 1 pill [ ]<br>*from Bottle B*
Day 22<br>AM: 2 pills [ ]<br>*from Bottle B*<br>PM: 2 pills [ ]<br>*from Bottle B*	Day 23<br>AM: 2 pills [ ]<br>*from Bottle B*<br>PM: 2 pills [ ]<br>*from Bottle B*	Day 24<br>AM: 2 pills [ ]<br>*from Bottle B*<br>PM: 2 pills [ ]<br>*from Bottle B*	Day 25<br>AM: 2 pills [ ]<br>*from Bottle B*<br>PM: 2 pills [ ]<br>*from Bottle B*	Day 26<br>AM: 2 pills [ ]<br>*from Bottle B*<br>PM: 2 pills [ ]<br>*from Bottle B*	Day 27<br>AM: 2 pills [ ]<br>*from Bottle B*<br>PM: 2 pills [ ]<br>*from Bottle B*	Day 28<br>AM: 2 pills [ ]<br>*from Bottle B*<br>PM: 2 pills [ ]<br>*from Bottle B*

NB (acute; Bottle A) → Placebo (maintenance; Bottle B)

Day 1<br>AM: 1 pill [ ]<br>*from Bottle A*<br>PM: 1 pill [ ]<br>*from Bottle A*	Day 2<br>AM: 1 pill [ ]<br>*from Bottle A*<br>PM: 1 pill [ ]<br>*from Bottle A*	Day 3<br>AM: 1 pill [ ]<br>*from Bottle A*<br>PM: 1 pill [ ]<br>*from Bottle A*	Day 4<br>AM: 1 pill [ ]<br>*from Bottle A*<br>PM: 1 pill [ ]<br>*from Bottle A*	Day 5<br>AM: 1 pill [ ]<br>*from Bottle A*<br>PM: 0 pills	Day 6<br>AM: 1 pill [ ]<br>*from Bottle A*<br>PM: 0 pills	Day 7<br>AM: 1 pill [ ]<br>*from Bottle A*<br>PM: 0 pills
Day 8<br>AM: 1 pill [ ]<br>*from Bottle B*<br>PM: 1 pill [ ]<br>*from Bottle B*	Day 9<br>AM: 1 pill [ ]<br>*from Bottle B*<br>PM: 1 pill [ ]<br>*from Bottle B*	Day 10<br>AM: 1 pill [ ]<br>*from Bottle B*<br>PM: 1 pill [ ]<br>*from Bottle B*	Day 11<br>AM: 1 pill [ ]<br>*from Bottle B*<br>PM: 1 pill [ ]<br>*from Bottle B*	Day 12<br>AM: 1 pill [ ]<br>*from Bottle B*<br>PM: 1 pill [ ]<br>*from Bottle B*	Day 13<br>AM: 1 pill [ ]<br>*from Bottle B*<br>PM: 1 pill [ ]<br>*from Bottle B*	Day 14<br>AM: 1 pill [ ]<br>*from Bottle B*<br>PM: 1 pill [ ]<br>*from Bottle B*
Day 15<br>AM: 2 pills [ ]<br>*from Bottle B*<br>PM: 1 pill [ ]<br>*from Bottle B*	Day 16<br>AM: 2 pills [ ]<br>*from Bottle B*<br>PM: 1 pill [ ]<br>*from Bottle B*	Day 17<br>AM: 2 pills [ ]<br>*from Bottle B*<br>PM: 1 pill [ ]<br>*from Bottle B*	Day 18<br>AM: 2 pills [ ]<br>*from Bottle B*<br>PM: 1 pill [ ]<br>*from Bottle B*	Day 19<br>AM: 2 pills [ ]<br>*from Bottle B*<br>PM: 1 pill [ ]<br>*from Bottle B*	Day 20<br>AM: 2 pills [ ]<br>*from Bottle B*<br>PM: 1 pill [ ]<br>*from Bottle B*	Day 21<br>AM: 2 pills [ ]<br>*from Bottle B*<br>PM: 1 pill [ ]<br>*from Bottle B*
Day 22<br>AM: 2 pills [ ]<br>*from Bottle B*<br>PM: 2 pills [ ]<br>*from Bottle B*	Day 23<br>AM: 2 pills [ ]<br>*from Bottle B*<br>PM: 2 pills [ ]<br>*from Bottle B*	Day 24<br>AM: 2 pills [ ]<br>*from Bottle B*<br>PM: 2 pills [ ]<br>*from Bottle B*	Day 25<br>AM: 2 pills [ ]<br>*from Bottle B*<br>PM: 2 pills [ ]<br>*from Bottle B*	Day 26<br>AM: 2 pills [ ]<br>*from Bottle B*<br>PM: 2 pills [ ]<br>*from Bottle B*	Day 27<br>AM: 2 pills [ ]<br>*from Bottle B*<br>PM: 2 pills [ ]<br>*from Bottle B*	Day 28<br>AM: 2 pills [ ]<br>*from Bottle B*<br>PM: 2 pills [ ]<br>*from Bottle B*

NB (acute; Bottle A) → NB (maintenance; Bottle B)

Day 1<br>AM: 2 pills [ ]<br>*from Bottle A*<br>PM: 2 pills [ ]<br>*from Bottle A*	Day 2<br>AM: 2 pills [ ]<br>*from Bottle A*<br>PM: 2 pills [ ]<br>*from Bottle A*	Day 3<br>AM: 2 pills [ ]<br>*from Bottle A*<br>PM: 2 pills [ ]<br>*from Bottle A*	Day 4<br>AM: 2 pills [ ]<br>*from Bottle A*<br>PM: 2 pills [ ]<br>*from Bottle A*	Day 5<br>AM: 2 pills [ ]<br>*from Bottle A*<br>PM: 2 pills [ ]<br>*from Bottle A*	Day 6<br>AM: 2 pills [ ]<br>*from Bottle A*<br>PM: 2 pills [ ]<br>*from Bottle A*	Day 7<br>AM: 2 pills [ ]<br>*from Bottle A*<br>PM: 2 pills [ ]<br>*from Bottle A*
Day 8<br>AM: 2 pills [ ]<br>*from Bottle B*<br>PM: 2 pills [ ]<br>*from Bottle B*	Day 9<br>AM: 2 pills [ ]<br>*from Bottle B*<br>PM: 2 pills [ ]<br>*from Bottle B*	Day 10<br>AM: 2 pills [ ]<br>*from Bottle B*<br>PM: 2 pills [ ]<br>*from Bottle B*	Day 11<br>AM: 2 pills [ ]<br>*from Bottle B*<br>PM: 2 pills [ ]<br>*from Bottle B*	Day 12<br>AM: 2 pills [ ]<br>*from Bottle B*<br>PM: 2 pills [ ]<br>*from Bottle B*	Day 13<br>AM: 2 pills [ ]<br>*from Bottle B*<br>PM: 2 pills [ ]<br>*from Bottle B*	Day 14<br>AM: 2 pills [ ]<br>*from Bottle B*<br>PM: 2 pills [ ]<br>*from Bottle B*
Day 15<br>AM: 2 pills [ ]<br>*from Bottle B*<br>PM: 2 pills [ ]<br>*from Bottle B*	Day 16<br>AM: 2 pills [ ]<br>*from Bottle B*<br>PM: 2 pills [ ]<br>*from Bottle B*	Day 17<br>AM: 2 pills [ ]<br>*from Bottle B*<br>PM: 2 pills [ ]<br>*from Bottle B*	Day 18<br>AM: 2 pills [ ]<br>*from Bottle B*<br>PM: 2 pills [ ]<br>*from Bottle B*	Day 19<br>AM: 2 pills [ ]<br>*from Bottle B*<br>PM: 2 pills [ ]<br>*from Bottle B*	Day 20<br>AM: 2 pills [ ]<br>*from Bottle B*<br>PM: 2 pills [ ]<br>*from Bottle B*	Day 21<br>AM: 2 pills [ ]<br>*from Bottle B*<br>PM: 2 pills [ ]<br>*from Bottle B*
Day 22<br>AM: 2 pills [ ]<br>*from Bottle B*<br>PM: 2 pills [ ]<br>*from Bottle B*	Day 23<br>AM: 2 pills [ ]<br>*from Bottle B*<br>PM: 2 pills [ ]<br>*from Bottle B*	Day 24<br>AM: 2 pills [ ]<br>*from Bottle B*<br>PM: 2 pills [ ]<br>*from Bottle B*	Day 25<br>AM: 2 pills [ ]<br>*from Bottle B*<br>PM: 2 pills [ ]<br>*from Bottle B*	Day 26<br>AM: 2 pills [ ]<br>*from Bottle B*<br>PM: 2 pills [ ]<br>*from Bottle B*	Day 27<br>AM: 2 pills [ ]<br>*from Bottle B*<br>PM: 2 pills [ ]<br>*from Bottle B*	Day 28<br>AM: 2 pills [ ]<br>*from Bottle B*<br>PM: 2 pills [ ]<br>*from Bottle B*
