# Supplementary material for: Naltrexone plus bupropion combination medication maintenance treatment for binge-eating disorder following successful acute treatments: randomized double-blind placebo-controlled trial
Source: Psychol Med. 2023 Jun 27;53(16):7775–84. doi: 10.1017/S0033291723001800 (PMC10751383; doi:10.1017/S0033291723001800)
Supplement: Grilo et al. supplementary material 2 — Grilo et al. supplementary material [file S0033291723001800sup002.docx]

**Supplemental Material Text**

**Double-blinded Re-Randomization Pharmacotherapy Protocol**

Our re-randomization pharmacotherapy protocol utilized a double-blinded down-titration of the initial double-blind pharmacotherapy following the acute treatment followed immediately by a double-blind up-titration of maintenance psychopharmacotherapy. This double-blind re-randomization protocol, intended to reduce potential confounds attributable to unblinding, is summarized below. Four **Tables**, which follow this overview, provide specific details about the double-blinded re-randomization protocol that took into account the assigned medication (naltrexone/bupropion or placebo) from the acute trial.

To maintain the double blind, all participants were given two pill bottles labeled “bottle A” and “bottle B” and a sealed envelope with directions for how many pills to take from each bottle on each day for four weeks. Dosing directions varied by acute and maintenance phase randomization assignment.

There were five participants categorized as responders to the acute treatment who had required reduced daily dosage of the study medication during the acute trial due to reported side effects. These five participants did not exceed the maximum dosing from the acute trial during the subsequent maintenance trial.
