# Supplementary material for: Naltrexone plus bupropion combination medication maintenance treatment for binge-eating disorder following successful acute treatments: randomized double-blind placebo-controlled trial
Source: Psychol Med. 2023 Jun 27;53(16):7775–84. doi: 10.1017/S0033291723001800 (PMC10751383; doi:10.1017/S0033291723001800)
Supplement: Grilo et al. supplementary material 3 — Grilo et al. supplementary material [file S0033291723001800sup003.docx]

**SUPPLEMENTAL TABLE 1.** Adverse Events (frequency) assessed systematically at month one

of the Stage 2 maintenance treatment (among responders to initial Stage 1 acute treatments).

__________________________________________________________________

|  | Placebo | Naltrexone/bupropion |  |  |
| --- | --- | --- | --- | --- |
| All-Cause Mortality | 0/34 (0%) | 0/32 (0%) |  |  |
| Serious Adverse Events | 0/34 (0%) | 0/32 (0%) |  |  |
| Hypertensive Crisis | 0/34 (0%) | 0/32 (0%) |  |  |
| Other (Not Including Serious) Adverse Events | | |  |  |
| Constipation | 5/34 (14.7%) | 10/32 (31.3%) |  | |
| Diarrhea | 5/34 (14.7%) | 2/32 (6.3%) |  | |
| Nausea | 3/34 (8.8%) | 8/32 (25.0%) |  | |
| Vomiting | 3/34 (8.8%) | 3/32 (9.4%) |  | |
| Dizziness | 4/34 (11.8%) | 4/32 (12.5%) |  | |
| Dry Mouth | 3/34 (8.8%) | 10/32 (31.3%) |  | |
| Headache | 4/34 (11.8%) | 9/32 (28.1%) |  | |
| Insomnia | 9/34 (26.5%) | 7/32 (21.9%) |  | |
| Anxiety | 6/34 (17.7%) | 2/32 (6.3%) |  | |

_____________________________________________________________________

Note: Adverse (side effect) events reported by >5% of participants in either naltrexone/bupropion or placebo treatment conditions during systematic assessment at month one during this maintenance trial. These adverse event data are included in the clinicaltrials.gov record (NCT03047005). We report side effects experienced by >5% of participants in either arm.
