# Supplementary material for: Naltrexone plus bupropion combination medication maintenance treatment for binge-eating disorder following successful acute treatments: randomized double-blind placebo-controlled trial
Source: Psychol Med. 2023 Jun 27;53(16):7775–84. doi: 10.1017/S0033291723001800 (PMC10751383; doi:10.1017/S0033291723001800)
Supplement: Grilo et al. supplementary material 4 — Grilo et al. supplementary material [file S0033291723001800sup004.docx]

**SUPPLEMENTAL TABLE 2.** Secondary clinical measures by treatment conditions.

_____________________________________________________________________________

|  | Placebo | | | Naltrexone/Bupropion | | |
| --- | --- | --- | --- | --- | --- | --- |
|  | n=34 | | | n=32 | | |
|  | n | M | SD | n | M | SD |
| EDE Global Score |  |  |  |  |  |  |
| Pre-Treatment | 34 | 1.63 | 0.80 | 32 | 1.57 | 0.73 |
| Post-Treatment | 28 | 1.63 | 0.88 | 29 | 1.46 | 0.97 |
| *Change* | 28 | 0.11 | 0.87 | 29 | -0.09 | 0.90 |
| BDI-II Depression Score |  |  |  |  |  |  |
| Pre-Treatment | 34 | 5.41 | 5.74 | 31 | 6.39 | 6.90 |
| Post-Treatment | 28 | 6.71 | 11.48 | 28 | 4.36 | 5.49 |
| *Change* | 28 | 1.68 | 7.88 | 27 | -1.89 | 6.06 |
| TFEQ Restraint Score |  |  |  |  |  |  |
| Pre-Treatment | 34 | 11.68 | 4.33 | 31 | 12.71 | 4.92 |
| Post-Treatment | 27 | 10.93 | 4.82 | 28 | 12.75 | 4.26 |
| *Change* | 27 | -0.15 | 3.19 | 27 | 0.00 | 3.57 |
| TFEQ Disinhibition Score |  |  |  |  |  |  |
| Pre-Treatment | 34 | 8.41 | 3.64 | 31 | 9.10 | 3.79 |
| Post-Treatment | 27 | 10.04 | 3.11 | 28 | 8.75 | 4.10 |
| *Change* | 27 | 1.67 | 2.83 | 27 | -0.19 | 3.49 |
| TFEQ Hunger Score |  |  |  |  |  |  |
| Pre-Treatment | 34 | 4.82 | 3.71 | 31 | 5.94 | 3.41 |
| Post-Treatment | 27 | 5.70 | 3.27 | 28 | 5.39 | 3.62 |
| *Change* | 27 | 0.93 | 3.35 | 27 | 0.00 | 2.99 |
| FCI Score |  |  |  |  |  |  |
| Pre-Treatment | 34 | 0.97 | 0.53 | 31 | 1.04 | 0.61 |
| Post-Treatment | 27 | 1.00 | 0.50 | 28 | 0.87 | 0.53 |
| *Change* | 27 | 0.05 | 0.35 | 27 | -0.21 | 0.65 |
| PFS Score |  |  |  |  |  |  |
| Pre-Treatment | 34 | 38.44 | 15.38 | 31 | 40.48 | 11.89 |
| Post-Treatment | 27 | 40.11 | 13.11 | 28 | 40.50 | 14.62 |
| *Change* | 27 | 2.04 | 10.73 | 27 | 0.26 | 11.76 |
| Cholesterol Total |  |  |  |  |  |  |
| Pre-Treatment | 34 | 192.09 | 27.96 | 32 | 194.28 | 36.53 |
| Post-Treatment | 27 | 191.78 | 30.61 | 28 | 195.57 | 33.26 |
| *Change* | 27 | -0.19 | 20.79 | 28 | -0.79 | 30.49 |
| HDL |  |  |  |  |  |  |
| Pre-Treatment | 34 | 51.91 | 12.61 | 32 | 55.13 | 13.57 |
| Post-Treatment | 27 | 52.48 | 13.08 | 28 | 60.86 | 14.22 |
| *Change* | 27 | 1.26 | 6.53 | 28 | 4.61 | 7.62 |
| LDL |  |  |  |  |  |  |
| Pre-Treatment | 34 | 118.68 | 24.59 | 32 | 118.09 | 31.94 |
| Post-Treatment | 27 | 118.30 | 26.80 | 28 | 115.00 | 29.74 |
| *Change* | 27 | -0.81 | 20.17 | 28 | -4.36 | 26.44 |
| HbA1c |  |  |  |  |  |  |
| Pre-Treatment | 33 | 5.35 | 0.33 | 32 | 5.36 | 0.43 |
| Post-Treatment | 27 | 5.45 | 0.45 | 28 | 5.31 | 0.35 |
| *Change* | 27 | 0.09 | 0.22 | 28 | -0.11 | 0.17 |

Note: Descriptive statistics for the secondary clinical outcomes.

n = number; M = mean; SD = standard deviation. EDE = Eating Disorder Examination interview;

BDI-II = Beck Depression Inventory – II (Beck at al. 1998); TFEQ = Three Factor Eating Questionnaire (Stunkard & Messick, 1985); FCI = Food Craving Inventory (White & Grilo, 2005); PFS = Power of Food Scale Cappelleri et al., 2009); HDL = high-density lipoprotein; LDL = low-density lipoprotein; HbA1c = glycated hemoglobin A1c level.

**References:**

Beck, A.T., Steer, R., & Garbin, M. (1998). Psychometric properties of the Beck Depression Inventory: 25 years of evaluation. *Clinical Psychology Review, 8*, 77-100.

Cappelleri, J.C., Bushmakin, A.G., Gerber, R.A., et al. (2009). Evaluating the Power of Food Scale in obese subjects and a general sample of individuals: development and measurement properties. *International Journal of Obesity, 33*, 913-22.

Stunkard, A.J., & Messick, S. (1985). The three-factor eating questionnaire to measure dietary restraint, disinhibition, and hunger. *Journal of Psychosomatic Research, 29*, 71-83.

White, M.A., & Grilo, C.M. (2005). Psychometric properties of the Food Craving Inventory among obese patients with binge eating disorder. *Eating Behaviors, 6*, 239-245.
